# Zika Virus: An Emerging Worldwide Threat

**DOI:** 10.3389/fmicb.2017.01417

**Published:** 2017-07-26

**Authors:** Irfan A. Rather, Jameel B. Lone, Vivek K. Bajpai, Woon K. Paek, Jeongheui Lim

**Affiliations:** ^1^Department of Biotechnology, Daegu University Gyeongsan, South Korea; ^2^Department of Applied Microbiology and Biotechnology, School of Biotechnology, Yeungnam University Gyeongsan, South Korea; ^3^National Science Museum, Ministry of Science, ICT and Future Planning Daejeon, South Korea

**Keywords:** ZIKV, disease, infection, vaccines, diagnosis

## Abstract

ZIKA virus (ZIKV) poses a severe threat to the world. Recent outbreaks of ZIKV after 2007 along with its quick transmission have made this virus a matter of international concern. The virus shows symptoms that are similar to those caused in the wake of dengue virus (DENV) and other flaviviruses, which makes it difficult to discern the viral infection. Diagnosis is further complicated as the virus cross-reacts with antibodies of other viruses. Currently, molecular diagnosis of the virus is being performed by RT-PCR and IgM-captured enzyme-linked immunosorbent assay (MAC-ELISA). The real brunt of the virus is, however, borne by children and adults alike. Case studies of the ZIKV outbreaks in the French Polynesia and other places have suggested that there is a close link between the ZIKV and Gullian-Barre syndrome (GBS). The GBS has closely followed in areas facing ZIKV outbreaks. Although solid evidence is yet to emerge, clinical data integration has revealed a large number of ZIKV patients having GBS. Moreover, the amniotic fluids, blood cord, and miscarriage tissues of mothers have been detected with ZIKV, which indicates that the virus either gets transferred from mother to fetus or seeks direct entry in the fetus, causing microcephaly and other brain anomalies in the newborn babies. Studies on mice have confirmed the link between the ZIKV infection during pregnancy and microcephaly in babies. Reports have highlighted the sexual transmission of the ZIKV, as it has been detected in the semen and saliva of affected persons. The intensity with which the ZIKA is spreading can collapse the health sector of several countries, which are poor. A comprehensive strategy is a need of an hour to combat this virus so as to prevent its transmission and avert the looming threat. At the same time, more research on the cure of the ZIKV is imperative.

## Introduction

ZIKA virus (ZIKV) belongs to the family of flaviviruses (Weaver, 2017a) that entails other viruses, such as yellow fever, dengue, Japanese encephalitis, and West Nile. The virus was first isolated from a rhesus monkey that was being used as one of the sentinel animals in a research program, which covered yellow fever. Soon after, the virus followed to infect humans in Nigeria in 1954 (Schuler-Faccini et al., 2017). It is transmitted through the arthropod vectors having serological overlapping with viruses like dengue virus (DENV) and West Nile Virus (Korhonen et al., 2016). Following the infection in 1954, there were about fourteen documented cases of the ZIKV prior to 2007 (Weaver, 2017b). The virus was only present in Asia and Africa and did not cause any major outbreaks. However, 2007 and 2013 saw two major outbreaks of ZIKV, reported from the Pacific Island of Yap and French Polynesia, respectively (Hall, 2017). The virus quickly spread to the American continent and 33 countries of America were hit by March 2016 (Hennessey et al., 2016). The symptoms were characterized by mild fever, arthralgia, conjunctivitis, and rash. The initial reports were of the opinion that ZIKV can cause mild febrile illness. The Brazilian cases of ZIKV, however, were reportedly linked to fetal microcephaly of ZIKV-infected mothers (Mlakar et al., 2016). Simultaneously, several patients of the ZIKV in French Polynesia showed neurological symptoms such as Guillain-Barreì syndrome (GBS) (Ioos et al., 2014) These reports along with the speedy spread of the virus were indications of the latent disorders associated with the ZIKV and its potential of becoming a global threat.

## Zika Virus Transmission

**FIGURE 1:**
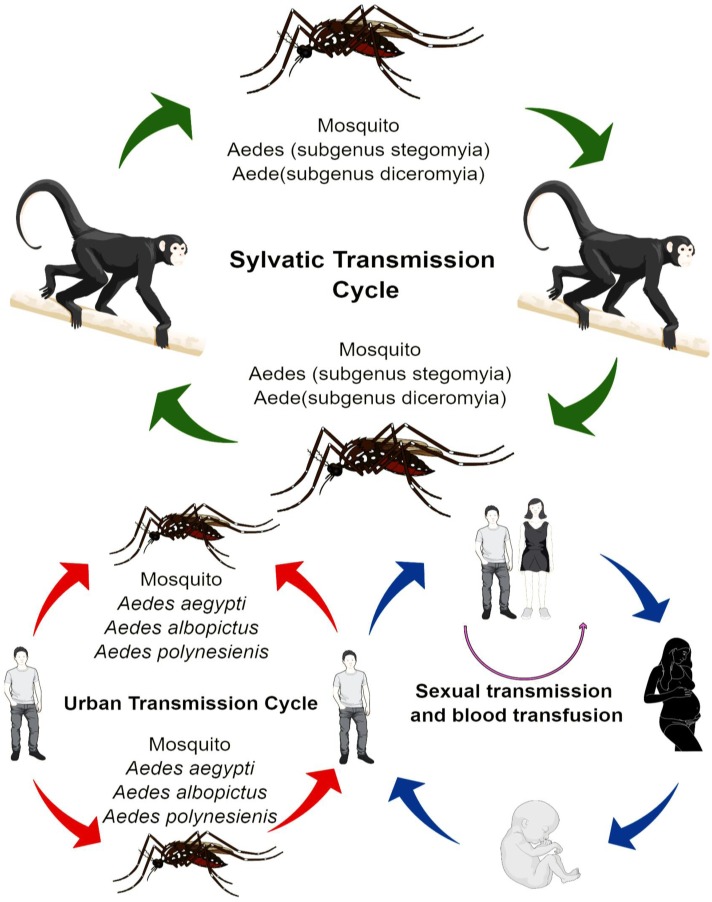
Transmission of ZIKA.

The transmission of ZIKV shows a high degree of variance. In Africa, the virus has adopted the sylvatic transmission cycle mode, which involves various species of Aedes mosquitoes and non-human primates, such as rhesus monkeys (**Figure 1**). Whereas, in the Asia, the sylvatic transmission cycle of ZIKV is yet to be reported (Diallo et al., 2014), where the ZIKV has adopted the transmission passages from the human-mosquito, and human-human transmission cycle. The most widely common vectors of ZIKA are mosquitoes from stegomyia and diceromyia sub-genera of Aedes, *Aedes africanus* and *A. furcifer*. *A. aegypti* and *A. albopictus* have been the primary vectors for majority of the ZIKV outbreaks (Ciota et al., 2017). However, in case of Yap, and Polynesia outbreaks, the *A. hensilli* and *A. polynesiensis* were the vectors of ZIKV, respectively (Musso et al., 2014). *A. aegypti* and *A. albopictus* are being considered as vectors with low vectorial competence (Santos and Meneses, 2017); however, with high vectorial capability, where low vectorial competence reduces the ability of the mosquito to acquire and transmit the ZIKV to other susceptible hosts. High vectorial capability, however, increases the efficiency of arthropods in transmitting the virus and is based on the number of bites, its longevity, and the population density of the mosquitoes among other factors. The high vectorial capability of *A. aegypti* and *A. albopictus* is attributed to many factors, such as close imperceptible bite and close association with humans (Chouin-Carneiro et al., 2016). Distribution of *A. aegypti* and *A. albopictus* is also a significant factor in the transmission of ZIKV. Moreover, there are other mosquito species, which could serve as a mode of transmission, fortunately, however, their vectorial capacity is remarkably low, and thus prevents further exacerbation of ZIKV problem (Diallo et al., 2014).

## Non-Mosquito Transmission

There are adequate reports that ZIKV has the capability to be transmitted from a mother to her fetus during the pregnancy. Virus particles and RNA were detected in the amniotic fluid of fetus (Calvet et al., 2016). Additionally, the ZIKV viral antigens also marked the placenta and miscarriage tissues of infected mothers (Meaney-Delman et al., 2016). Recent study by Pagani et al. (2017) reported that primary human endometrial stromal cells are greatly permissive to ZIKV infection and supports its *in vitro* replication. Perinatal transmission of ZIKV was also reported in French Polynesia outbreak. A study also suggested that routes of perinatal transmission are mainly transplacental, breastfeeding, close contact between mother and baby during delivery (Colt et al., 2017). ZIKV sequences have been detected in the semen 62 days after the onset of symptoms. The data available hint the possible transmission of the virus through vaginal and oral sex (Hills et al., 2016; Russell et al., 2016). Nonetheless, transmission role of other biological fluid, such as pre-ejaculation semen, and saliva transmission cannot be ruled out (Cowper’s gland). Another non-mosquito transmission could be the blood transfusion (Bierlaire et al., 2017). During French Polynesia 3 of donated blood samples were tested positive for ZIKV.

The use of an animal model to study ZIKA infection is crucial for fundamental studies and development of effective interventions. In recent years, significant efforts have been made to develop mouse and non-human primate models to study ZIKA infection (Dudley et al., 2016). Subcutaneous inoculation of ZIKA in non-human primate resulted in the development of fetal brain lesions (Waldorf et al. (2016) and neonatal pigs were found highly susceptible to ZIKA infection (Darbellay et al., 2017). Also, a parallel study reported that subcutaneous administration of Asian-lineage ZIKA in pregnant rhesus macaques resulted in highly efficient maternal-fetal ZIKA transmission (Nguyen et al., 2017). The results propose that maternal-fetal ZIKA transmission could be frequent in human pregnancies. Another study by Haddow et al. (2017) reported high infection rates among adult macaques after intra-vaginal or intra-rectal inoculation. It proposes that ZIKA infection by sexual intercourse could increase the chances of spread of ZIKA in regions where the virus has not been reported.

## Virology

**FIGURE 2:**
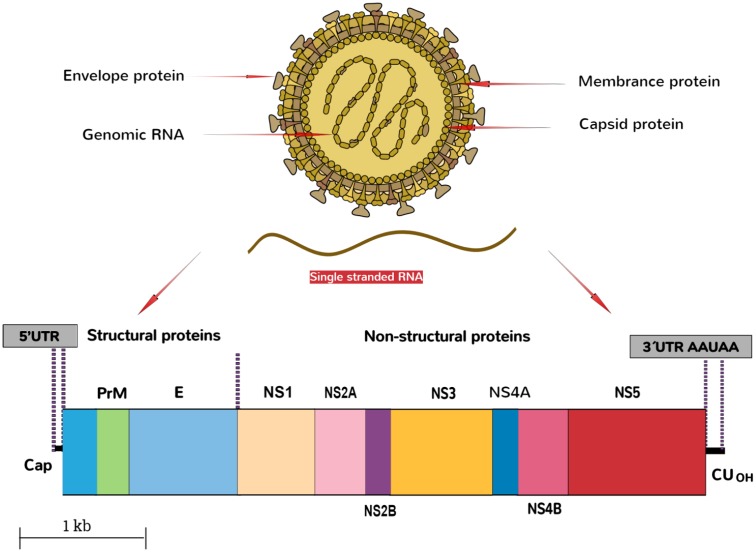
Schematic representation of ZIKA particle and its genome organization.

The ZIKV belongs to the family, Flaviviridae, genus Flavivirus. The virus is an arthropod-borne virus or arbovirus. The infectious particle of the virus known as virion is surrounded by a lipid membrane embedded by the viral membrane protein (Protein M), and envelope protein (Protein E). The virions are icosahedral, enveloped and contain a single-stranded, non-segmented RNA genome of the positive strand (Cortese et al., 2017) that encodes seven non-structural and three structural proteins. These are expressed as a singular polyprotein, which undergoes cleavage (White et al., 2016). The virion seeks the entry into the host cell by clathrin-mediated endocytosis. Removal of the envelope is followed by the disruption of nucleocapsid, and the genome is released into the cytoplasm. The genomic RNA of ZIKV then replicates in the cytoplasm of the infected host cells. The genome of the virus is translated by translational apparatus of the host cell, and results into the formation of single polyprotein that is proteolytically cleaved into the individual viral proteins, PreM, C, and non-structural proteins NS1 to NS5 (**Figure 2**).

## Acute Febrile Illness and Neurological Complications

The diagnosis of undifferentiated febrile illness is challenging as its relevant symptoms overlap with other common infections (Maria et al., 2013). The adults are more vulnerable to acute febrile illness that is marked by popular rashes, arthritis, non-purulent conjunctivitis, headache, vomiting, and myalgia (Zammarchi et al., 2015). Numerous case studies in the French Polynesia have determined the relationship between GBS and ZIKV (De Oliveira et al., 2017), where GBS is a disorder of the immune system that is characterized by sensory abnormalities, autonomic dysfunction, and weakness due to nerve root orperipheral damage (Dirlikov et al., 2017). During the ZIKV outbreaks, record numbers of patients were found to have GBS (Uncini et al., 2016) Subcutaneous bleeding and hematospermia were other symptoms associated with GBS followed by ZIKV syndrome (Lyle et al., 2016). According to European Center for Disease Prevention and Control [ECDC] (2016), the data of French Polynesia outbreak hints a possible link between GBS and ZIKV, which was later validated by French Ministry of Health in Martinique. In neonates, the neurological complications are on the rise unabated. Microcephaly in Brazil has dramatically increased in the last 3 years. This alarming situation has compelled the government to declare it as a national health emergency, which was further reciprocated by WHO (2016) (Gulland, 2016). Rasmussen et al. (2016) have reviewed and evaluated the available data on the ZIKV infection during pregnancy and concluded that there was a causal relation between ZIKV infection and microcephaly along with other serious brain anomalies. Other studies have also shown evidences from the available reports of clinical data that proves the causal relation between ZIKV and microcephaly due to the viral infection throughout the length of pregnancy leading to consistent cases of birth defects (Mlakar et al., 2016). Vermillion et al. (2017) also conducted a study on the pregnant mice by inoculating the uterine wall of the immunocompetent mice to understand the transplacental transmission. Results showed that the ZIKV infection during pregnancy caused several fetal abnormalities, which included microcephaly, intrauterine growth restriction, and enlarged ventricle. These recent studies have established a direct link of microcephaly with the ZIKV infection in a pregnant mother.

## Diagnosis

The diagnosis of ZIKA is primarily being done either by RT-PCR-based tests or neutralization assay where immune globin IgM is detected by IgM-captured enzyme-linked immunosorbent assay (MAC-ELISA) (Tappe et al., 2014). Viral nucleic acid can be traced in the serum within 1 week after the onset of clinical illness. However, there is no solid evidence about the response and persistence of IgM antibody-mediated neutralization. The data of other mosquito-borne flaviviruses suggest that formation of IgM antibody takes places within 10 days after onset of clinical illness and can persist for more than 2 months (Kalra et al., 2014). The greatest challenge in the diagnosis of ZIKV is the cross-reactivity of the flaviviruses. Sometimes ZIKV infected patients evoke positive MAC-ELISA for DENV, and other related viruses (Tauro et al., 2016). The differentiation of closely related antibodies of closely related viruses could overcome by plaque reduction neutralization test (PRNT); however, high cost and labor-intensive reasons do not make it an ideal choice for clinicians (Roehrig et al., 2008). Moreover, the problem of cross-reactivity gets further compounded by the Hoskins effect (Original antigenic sin) (Lanciott et al., 2016). Due to the vaccination or natural infection of closely related flaviviruses, the memory response is more robust to the former than a freshly entered virus. Even this problem could not be solved by high accuracy of PRNT (Mohammed et al., 2012). Furthermore, the saliva and serum of the patients were subjected to RT-PCR and were found sensitive in detection (Rozé et al., 2016). There is still no reliable test for diagnosis of prenatal ZIKV infection, although RT-PCR can be performed on amniotic fluid, and other tissues like blood chod, and so on.

## Prevention and Control

At present, there is no vaccine available in the market against ZIKV, thus it is imperative that coordinated, multidimensional, and comprehensive strategies are made to deal with any eventuality. Like other flavivirus infections, the treatment for ZIKV is entirely based on the symptoms. The primary strategy to adopt and to deal with ZIKA outbreak is restricting the vectorial capacity of *A. aegypti* viruses by eliminating their breeding sites, application of larvacides, use of mosquito repellents, bed nets, avoiding sleep in the day, and to maintain the green and clean environment (Banks et al., 2014). The sexual transmission of ZIKV could be prevented by avoiding the unprotected sex, and sexual contact with the persons who are vulnerable of getting infected or traveling from virus prone areas (Petersen et al., 2016). Interference at the genetic level of bacteria like *Wolbachia* could be very beneficial in restricting the transmission of ZIKV (Weaver, 2013). The entry of *Wolbachia* bacteria in a cycling pool of vector will have a cascading effect as the population of *Wolbachia* carrying mosquitoes will expand with each cycle of reproduction (Nguyen et al., 2015). Moreover, the use of genetically modified mosquito strains has been found effective in DENV, and thus are likely to be used against ZIKV as well. *A. aegypti* OX513A is a genetically modified (GM) strain, leading to a reduction of the local population of *A. aegypti*. The GM male mosquitoes can mate with wild type females and thus can eliminate them (Alphey and Alphey, 2014). In order to prevent the mother-fetus transmission, pregnant women should avoid unnecessary travels to the ZIKV affected areas. In addition, the government must alert the people who are traveling to affected areas.

## Future Outlook

The cross-reactivity of serological assays and mild symptoms of ZIKV make it difficult for scientists to gauge it at the preliminary stage. The risk of ZIKV multiplies in the areas where DENV is an epidemic as both belong to the family of flavivirus. The emergence of ZIKV is still an enigma for the scientific community; however, a general trend has been observed across the globe in which transmission of DENV, and chikungunya infections are followed by ZIKV infection (Musso et al., 2015). The simultaneous outbreaks of DENV, and other flaviviruses give a kind of refuge to ZIKV as it is being either misdiagnosed or getting undiagnosed given the reason of lack of standard molecular diagnostic test. Another important aspect of ZIKV endemic transmission could be the change of virulence by *A. aegypti*. The expansion of urbanization and transmission of flavivirus have been very much proportional. The greatest challenge before the scientific community is to save the incoming generation (newborn babies) from the onslaught of ZIKV infection. The relationship between the GBS in French Polynesia is very alarming and could further exacerbate child healthcare in the world. The rise in microcephaly and GBS in the aftermath of ZIKV outbreaks is a serious matter of concern and requires proper treatment so that the disease does not nip out tulips (children) in the bud. In order to develop an effective strategy against ZIKV, and other flaviviruses, there is a need to systematically identify, and address the loopholes in virus research. Diagnostic assays for the ZIKV are garnering intense interest and there is hope that in the near future promising strategies for the improved diagnostics of ZIKV will translate into therapeutic and preventive tools. Development of animal models for fetus development would further deepen the understanding of the transmission of ZIKV from mother to fetus. The present clinical data must be integrated, and new reliable, affordable molecular diagnostic tests must be developed to cope up with ZIKA endemic. Climate change is also a contributive factor in the transmission of many flaviviruses. New reports have outlined that rising temperature of earth suits the breeding patterns of mosquitoes. Globalization of trade is another route for the transmission of the virus specifically due to the rubber tires of transportation vehicles serving as breeding sites for mosquitoes. For the time being, an accurate vaccine for the treatment of the ZIKV is unavailable, therefore, there is little that can be done to reverse the adverse effects that the virus is having on the babies in the form of microcephaly and GBS. New and efficient ways of vector controlling mechanism must be introduced, and influence of environmental factors on ZIKV emergence should be understood in an elaborate manner. Although herd immunity can slow the transmission rate to some extent, it cannot be a replacement for the appropriate vaccination. Hence, it is imperative that efforts to develop therapeutic tools like a vaccine against ZIKV must continue unabated to develop a cure as soon as possible.

## Conclusion

Zika virus is a threat of international concern that requires immediate attention. Success against this threat can only be achieved by continuing the extensive research regarding this virus to be able to find an appropriate vaccine to tackle with the menace. The relationship, mode of action, and transmission among DENV, chikungunya and ZIKV infections should also be verified. A breakthrough in either of the mentioned viruses could be a milestone in the history of medicine as all of these viruses have a high degree of genetic similarity because they belong to the family of flavivirus. Until then, a comprehensive multidimensional strategy must be employed to strengthen public awareness in this regard and control the spread of the ZIKV by curbing its transmission through sexual intercourse, travel to affected areas and global trade.

## Author Contributions

IR and JBL wrote the paper. VB design the paper. WP and JL did the critical analysis and approved the paper.

## Conflict of Interest Statement

The authors declare that the research was conducted in the absence of any commercial or financial relationships that could be construed as a potential conflict of interest.

## References

[B1] Alphey L. Alphey N. (2014). Five things to know about genetically modified (GM) insects for vector control. *PLoS Pathog.* 10:e1003909 10.1371/journal.ppat.1003909 PMC3946353 24603810

[B2] Banks S. D. Murray N. Wilder-Smith A. Logan J. G. (2014). Insecticide-treated clothes for the control of vector-borne diseases: a review on effectiveness and safety. *Med. Vet. Entomol.* 28 14–25. 10.1111/mve.12068 24912919

[B3] Bierlaire D. Mauguin S. Broult J. Musso D. (2017). Zika virus and blood transfusion: the experience of French Polynesia. *Transfusion* 57 729–733. 10.1111/trf.14028 28185278

[B4] Calvet G. Aguiar R. S. Melo A. S. Sampaio S. A. de Filippis I. Fabri A. (2016). Detection and sequencing of Zika virus from amniotic fluid of fetuses with microcephaly in Brazil: a case study. *Lancet Infect. Dis.* 16 253–260. 10.1016/S1473-3099(16)00095-5 26897108

[B5] Chouin-Carneiro T. Vega-Rua A. Vazeille M. Yebakima A. Girod R. Goindin D. (2016). Differential susceptibilities of *Aedes aegypti* and *Aedes albopictus* from the Americas to Zika virus. *PLoS Negl. Trop. Dis.* 10:e0004543 10.1371/journal.pntd.0004543 PMC4777396 26938868

[B6] Ciota A. Bialosuknia S. Ehrbar D. Kramer L. (2017). Vertical transmission of zika virus by *Aedes aegypti* and Ae. albopictus Mosquitoes. *Emerg. Infect. Dis.* 23 880–882. 10.3201/eid2305.162041 28277199 PMC5403030

[B7] Colt S. Garcia-Casal M. Peña-Rosas J. Finkelstein J. Rayco-Solon P. Weise Prinzo Z. (2017). Transmission of Zika virus through breast milk and other breastfeeding-related bodily-fluids: a systematic review. *PLOS Negl. Trop. Dis.* 11:e0005528 10.1371/journal.pntd.0005528 PMC5398716 28394887

[B8] Cortese M. Goellner S. Acosta E. Neufeldt C. Oleksiuk O. Lampe M. (2017). Ultrastructural characterization of Zika virus replication factories. *Cell Rep.* 18 2113–2123. 10.1016/j.celrep.2017.02.014 28249158 PMC5340982

[B9] Darbellay J. Lai K. Babiuk S. Berhane Y. Ambagala A. Wheler C. (2017). Neonatal pigs are susceptible to experimental Zika virus infection. *Emerg. Microbes Infect.* 6 e6 10.1038/emi.2016.133 PMC5322322 28196970

[B10] De Oliveira W. Carmo E. Henriques C. Coelho G. Vazquez E. Cortez-Escalante J. (2017). Zika virus infection and associated neurologic disorders in Brazil. *New Engl. J. Med.* 376 1591–1593. 10.1056/nejmc1608612 28402236 PMC5544116

[B11] Diallo D. Sall A. A. Diagne C. T. Faye O. Faye O. Ba Y. (2014). Zika virus emergence in mosquitoes in southeastern Senegal, 2011. *PLoS ONE* 9:e109442 10.1371/journal.pone.0109442 PMC4195678 25310102

[B12] Dirlikov E. Kniss K. Major C. Thomas D. Virgen C. Mayshack M. (2017). Guillain-barré syndrome and healthcare needs during Zika virus transmission, rico puerto, 2016. *Emerg. Infect. Dis.* 23 134–136. 10.3201/eid2301.161290 27779466 PMC5176211

[B13] Dudley D. M. Aliota M. T. Mohr E. L. Weiler A. M. Lehrer-Brey G. Connor D. H. (2016). A rhesus macaque model of Asian-lineage Zika virus infection. *Nat. Commun.* 28 12204 10.1038/ncomms12204 PMC4931337 27352279

[B14] European Center for Disease Prevention and Control [ECDC] (2016). *Zika Virus Epidemic in the Americas: Potential Association with Microcephaly and Guillain-Barré syndrome*. Stockholm: ECDC.

[B15] Gulland A. (2016). Zika virus is a global public health emergency, declares WHO. *BMJ.* 352 i657 10.1136/bmj.i657 26839247

[B16] Haddow A. D. Nalca A. Rossi F. D. Miller L. J. Wiley M. R. Perez-Sautu U. (2017). High infection rates for adult macaques after intravaginal or intrarectal inoculation with Zika virus. *Emerg. Infect. Dis.* 23 8 10.3201/eid2308.170036 PMC5547779 28548637

[B17] Hall B. (2017). *The Emergence of Zika Virus: The Heron 2017* 1st Edn Vol. 7. Available at: http://greatbay.edu/sites/default/files/media/The-Heron-Vol7_2017.pdf\#page = 20

[B18] Hennessey M. Fischer M. Staples J. E. (2016). Zika virus spreads to new areas – region of the Americas, May 2015–January 2016. *MMWR Morb. Mortal. Wkly. Rep.* 65 55–58. 10.15585/mmwr.mm6503e1 26820163

[B19] Hills S. L. Russell K. Hennessey M. Williams C. Oster A. M. Fischer M. (2016). Transmission of Zika virus through sexual contact with travelers to areas of ongoing transmission — continental united states, 2016. *MMWR Morb. Mortal. Wkly. Rep.* 65 215–216. 10.15585/mmwr.mm6508e2 26937739

[B20] Ioos S. Mallet H. Leparc Goffart I. Gauthier V. Cardoso T. Herida M. (2014). Current Zika virus epidemiology and recent epidemics. *Méd. Mal. Infect.* 44 302–307. 10.1016/j.medmal.2014.04.008 25001879

[B21] Kalra S. Kelkar D. Galwankar S. C. Papadimos T. J. Stawicki S. P. Arquilla B. (2014). The emergence of *Ebola* as a global health security threat: from ‘lessons learned’ to coordinated multilateral containment efforts. *J. Glob. Infect. Dis.* 6 64–77. 10.4103/0974-777X.145247 PMC4265832 25538455

[B22] Korhonen E. M. Huhtamo E. Smura T. Kallio-Kokko H. Raassina M. Vapalahti O. (2016). Zika virus infection in a traveler returning from the Maldives, June 2015. *Euro Surveill.* 21 30107 10.2807/1560-7917.ES.2016.21.2.30107 26794427

[B23] Lanciott R. S. Lambert A. J. Holodniv M. Saavedra S. Signor L. (2016). Phylogeny of Zika virus in Western Hemisphere 2015. *Emerg. Infect. Dis.* 22 933–935. 10.3201/eid2205.160065 27088323 PMC4861537

[B24] Lyle R. P. Denise J. J. Ann M. P. Margaret A. H. (2016). Zika Virus. *N. Engl J. Med.* 374 1552–1563. 10.1056/NEJMra1602113 27028561

[B25] Maria R. C. Mary N. C. Sri R. H. Ismail I. H. M. H. Revathy N. Punnee P. (2013). Dengue and other common causes of acute febrile illness in asia: an active surveillance study in children. *PLoS Negl. Trop. Dis.* 7:e2331 10.1371/journal.pntd.0002331 PMC3723539 23936565

[B26] Meaney-Delman D. Hills S. L. Williams C. Galang R. R. Iyengar P. Hennenfent A. K. (2016). Zika virus infection among U.S. pregnant travelers – August 2015–February 2016. *MMWR Morb. Mortal. Wkly. Rep.* 65 211–214. 10.15585/mmwr.mm6508e1 26938703

[B27] Mlakar J. Korva M. Tul N. Popović M. Poljšak-Prijatelj M. Mraz J. (2016). Zika virus associated with microcephaly. *New Engl. J. Med.* 374 951–958. 10.1056/nejmoa1600651 26862926

[B28] Mohammed H. Tomashek K. M. Stramer S. L. Hunsperger E. (2012). Prevalence of antidengue immunoglobulin G antibodies among American Red Cross blood donors in Puerto Rico, 2006. *Transfusion* 52 6 10.1111/j.1537-2995.2011.03492.x 22224623

[B29] Musso D. Cao-Lormeau V. M. Gubler D. J. (2015). Zika virus: following the path of dengue and chikungunya? *Lancet* 386 243–244. 10.1016/S0140-6736(15)61273-9 26194519

[B30] Musso D. Nilles E. J. Cao-Lormeau V. M. (2014). Rapid spread of emerging Zika virus in the Pacific area. *Clin. Microbiol. Infect.* 20 595–596. 10.1111/1469-0691.12707 24909208

[B31] Nguyen S. M. Antony K. M. Dudley D. M. Golos T. G. (2017). Highly efficient maternal-fetal Zika virus transmission in pregnant rhesus macaques. *PLoS Pathog.* 13:e1006378 10.1371/journal.ppat.1006378 PMC5444831 28542585

[B32] Nguyen T. H. Nguyen H. L. Nguyen T. Y. Vu S. N. Tran N. D. Le T. N. (2015). Field evaluation of the establishment potential of wmelPop Wolbachia in Australia and Vietnam for dengue control. *Parasit. Vectors* 8 563 10.1186/s13071-015-1174-x PMC4625535 26510523

[B33] Pagani I. Ghezzi S. Ulisse A. Rubio A. Turrini F. Garavaglia E. (2017). Human endometrial stromal cells are highly permissive to productive infection by Zika virus. *Sci. Rep.* 7:44286 10.1038/srep44286 PMC5345097 28281680

[B34] Petersen E. E. Polen K. N. Meaney-Delman D. Ellington S. R. Oduyebo T. Cohn A. (2016). Update: interim guidance for health care providers caring for women of reproductive age with possible Zika virus exposure – united states, 2016. *MMWR Morb. Mortal. Wkly. Rep.* 65 315–322. 10.15585/mmwr.mm6512e2 27031943

[B35] Rasmussen S. Jamieson D. Honein M. Petersen L. (2016). Zika virus and birth defects — reviewing the evidence for causality. *New Engl. J. Med.* 374 1981–1987. 10.1056/nejmsr1604338 27074377

[B36] Roehrig J. T. Hombach J. Barrett A. D. (2008). Guidelines for plaque-reduction neutralization testing of human antibodies to dengue viruses. *Viral Immunol.* 21 123–132. 10.1089/vim.2008.0007 18476771

[B37] Rozé B. Najioullah F. Fergé J. L. Apetse K. Brouste Y. Cesaire R. (2016). Zika virus detection in urine from patients with Guillain-Barré syndrome on Martinique, January 2016. *Euro. Surveill.* 21 9 10.2807/1560-7917.ES.2016.21.9.30154 26967758

[B38] Russell K. Hills S. Oster A. Porse C. Danyluk G. Cone M. (2016). Male-to-female sexual transmission of Zika virus—united states, january–April 2016. *Clin. Infect. Dis.* 64 211–213. 10.1093/cid/ciw692 27986688

[B39] Santos J. Meneses B. (2017). An integrated approach for the assessment of the *Aedes aegypti* and *Aedes albopictus* global spatial distribution, and determination of the zones susceptible to the development of Zika virus. *Acta Trop.* 168 80–90. 10.1016/j.actatropica.2017.01.015 28111132

[B40] Schuler-Faccini L. Roehe P. Zimmer E. Quincozes-Santos A. de Assis A. Lima E. (2017). ZIKA Virus and neuroscience: the need for a translational collaboration. *Mol. Neurobiol.* 10.1007/s12035-017-0429-2 [Epub ahead of print]. 28185126

[B41] Tappe D. Rissland J. Gabriel M. Emmerich P. Gunther S. Held G. (2014). First case of laboratory-confirmed Zika virus infection imported into Europe, November 2013. *Euro Surveill.* 19 20685 10.2807/1560-7917.ES2014.19.4.20685 24507467

[B42] Tauro L. Bandeira A. Ribeiro G. Reis M. Pizarro C. Araujo K. (2016). Potential use of saliva samples to diagnose Zika virus infection. *J. Med. Virol.* 89 1–2. 10.1002/jmv.24696 27671098

[B43] Uncini A. Shahrizaila N. Kuwabara S. (2016). Zika virus infection and Guillain-Barré syndrome: a review focused on clinical and electrophysiological subtypes. *J. Neurol. Neurosurg. Psychiatry* 88 266–271. 10.1136/jnnp-2016-314310 27799296

[B44] Vermillion M. S. Lei J. Shabi Y. Baxter V. K. Crilly N. P. McLane M. (2017). Intrauterine Zika virus infection of pregnant immunocompetent mice models transplacental transmission and adverse perinatal outcomes. *Nat. Commun.* 8:14575 10.1038/ncomms14575 PMC5321801 28220786

[B45] Waldorf K. M. A. Stencel-Baerenwald J. E. Kapur R. P. Studholme C. Boldenow E. Vornhagen J. (2016). Fetal brain lesions after subcutaneous inoculation of Zika virus in a pregnant nonhuman primate. *Nat Med.* 22 1256–1259. 10.1038/nm.4193 27618651 PMC5365281

[B46] Weaver S. (2017a). C-102 Zika virus. *JAIDS J. Acquir. Immune Defic. Syndr.* 74 42 10.1097/01.qai.0000513827.78163.66

[B47] Weaver S. (2017b). Emergence of epidemic Zika virus transmission and congenital Zika syndrome: are recently evolved traits to blame? *Mbio* 8:e2063 10.1128/mbio.02063-16 PMC5225313 28074023

[B48] Weaver S. C. (2013). Urbanization and geographic expansion of zoonotic arboviral diseases: mechanisms and potential strategies for prevention. *Trends Microbiol.* 21 360–363. 10.1016/j.tim.2013.03.003 23910545 PMC5193003

[B49] White M. Wollebo H. David Beckham J. Tyler K. Khalili K. (2016). Zika virus: an emergent neuropathological agent. *Ann. Neurol.* 80 479–489. 10.1002/ana.24748 27464346 PMC5086418

[B50] WHO (2016). *Zika Virus Microcephaly and Guillain-Barré Syndrome* Vol. 17. Geneva: World Health Organization 1–12.

[B51] Zammarchi L. Stella G. Mantella A. Bartolozzi D. Tappe D. Günther S. (2015). Zika virus infections imported to Italy: clinical, immunological and virological findings, and public health implications. *J. Clin. Virol.* 63 32–35. 10.1016/j.jcv.2014.12.005 25600600

