# Corrigendum: Zika Virus: An Emerging Worldwide Threat

**DOI:** 10.3389/fmicb.2017.01740

**Published:** 2017-09-04

**Authors:** Irfan A. Rather, Jameel B. Lone, Vivek K. Bajpai, Woon K. Paek, Jeongheui Lim

**Affiliations:** ^1^Department of Applied Microbiology and Biotechnology, School of Biotechnology, Yeungnam University Gyeongsan, South Korea; ^2^Department of Biotechnology, Daegu University Gyeongsan, South Korea; ^3^National Science Museum, Ministry of Science, ICT and Future Planning Daejeon, South Korea

**Keywords:** ZIKV, disease, infection, vaccines, diagnosis

In the published article, affiliations 1 and 2 were switched. Affiliation 1 should be: Department of Applied Microbiology and Biotechnology, School of Biotechnology, Yeungnam University, Gyeongsan, South Korea. Affiliation 2 should be: Department of Biotechnology, Daegu University, Gyeongsan, South Korea.

The authors apologize for this error and state that this does not change the scientific conclusions of the article in any way.

## Conflict of interest statement

The authors declare that the research was conducted in the absence of any commercial or financial relationships that could be construed as a potential conflict of interest.

